# Efficacy of interleukin 10 gene hydrofection in pig liver vascular isolated ‘in vivo’ by surgical procedure with interest in liver transplantation

**DOI:** 10.1371/journal.pone.0224568

**Published:** 2019-11-05

**Authors:** Luis Sendra, María José Herrero, Eva María Montalvá, Inmaculada Noguera, Francisco Orbis, Ana Díaz, Rafael Fernández-Delgado, Rafael López-Andújar, Salvador F. Aliño

**Affiliations:** 1 Pharmacogenetics Unit, Instituto de Investigación Sanitaria La Fe, Valencia, Spain; 2 Gene Therapy Unit, Department of Pharmacology, Universitat de Valencia, Valencia, Spain; 3 Unit of Experimental Hepatology and Liver Transplantation, Instituto de Investigación Sanitaria La Fe, Valencia, Spain; 4 HPB Surgery and Transplant Unit, Hospital Universitario y Politécnico La Fe, Valencia, Spain; 5 SCSIE, Central Services of Experimental Support, Universitat de Valencia, Valencia, Spain; 6 Pediatrics Unit, Department of Pediatrics, Obstetrics and Gynecology, Universitat de Valencia, Valencia, Spain; 7 Clinical Pharmacology Unit, Hospital Universitario y Politécnico La Fe, Valencia, Spain; University of Alabama at Birmingham, UNITED STATES

## Abstract

**Aim:**

Liver transplantation is the only curative strategy for final stage liver diseases. Despite the great advances achieved during the last 20 years, the recipient immune response after transplantation is not entirely controlled. This results in high rates of acute cell rejection and, approximately, 10% of early mortality. Therapeutic treatment could be improved by efficiently transfecting genes that encode natural immunosuppressant proteins, employing safe procedures that could be transferred to clinical setting. In this sense, interleukin 10 plays a central role in immune tolerance response by acting at different levels.

**Methods:**

*hIL10* gene was hydrofected by retrograde hydrodynamic injection in pig liver with complete vascular exclusion mediated by an ‘in vivo’ surgical procedure. Levels of IL10 DNA, RNA and protein were determined within liver tissue 1 and 10 days after the injection and, more frequently, also the interleukin-10 protein in peripheral blood.

**Results:**

The procedure was safe for the animals and neither hemodynamic parameters nor liver function determinations showed relevant alterations. The hIL10 hydrofection in watertight liver mediated efficient gene transfer and this was transcribed and translated to protein, achieving up to 110 pg/ml of IL10 in peripheral blood. This value is close to that considered able to reduce the activity of TNFα by half (IL10 IC_50_ for TNFα = 124 pg/ml).

**Conclusions:**

Results of this work suggest that IL10 liver hydrofection with vascular exclusion in vivo is a safe and transferable procedure that mediates plasma protein levels with potential clinical interest in immune modulation after transplantation.

## Introduction

Liver transplantation is often the only treatment option for patients with otherwise non-treatable acute or fulminant liver disease or end-stage chronic liver disease. The main limitations of the liver transplantation are the scarce number of donors and the risk of organ rejection, what reduces the success of the intervention.

The great success of liver transplantation is mainly due, apart from the advances in surgical techniques and organ preservation strategies, to the special immune tolerance environment present within liver has played an invaluable role for recipient acceptance of the graft. In this regard, the development of immunosuppressant drugs and management improves patients’ immune balance. Despite substantial technological, medical and surgical advances, post-translation early mortality remains as high as 5–12% and acute cellular rejection has a high incidence (25–30%) within the first year post-transplantation [1]. Despite the acute cellular rejection is treated with corticosteroids, vascular and bile duct damage can occur. This indicates that the immediate immune response post-transplantation has not been completely controlled and should be improved for long-term management of transplanted patient. The liver is able to modulate immunity reactivity against alloantigens due to its continuous contact with nutrients and bacterial alloantigens proceeding from intestinal blood, shared through portal circulation [2]. This function is very relevant in humans’ interaction with environment. Since there are natural mechanisms of immune tolerance mediated by immunomodulatory cytokines, we hypothesize that effective transfer of genes encoding these tolerogenic cytokines to graft liver could contribute to improve the efficacy of transplant procedure in humans. Several genes encoding immunosuppressant proteins, such as IL-10 and TGF-β, and procedures (viral and non-viral) could be employed for this purpose, but the procedure safety and the gene expression efficacy must be guaranteed. In this study, we propose to evaluate whether a gene encoding the immunosuppressant cytokine IL-10 could be transferred by a safe non-viral gene transfer procedure (hydrofection) to the liver and achieve levels of protein expression (inhibitory concentration 50%, IC_50_) with potential clinical interest.

Interleukin 10 plays a relevant role in the control of immune activation response: suppresses the antigen presentation by specialized cells; inhibits the expression of proinflammatory cytokines [3] such as interferon γ, IL-2, IL-3, and GM-CSF. IL-10 also transforms naïve lymphocytes into regulatory T cells (Tregs) [4], which are mainly responsible for the immunosuppressant response [5] and can suppress different proinflammatory cells, including CD4+ T cells, CD8+ T cells, NKT cells, dendritic cells, monocytes/macrophages, B cells, and NK cells [6]. The presence of Tregs has proved to be crucial for setting up the graft tolerance mechanisms [7].

The aim of this work was to evaluate the potential expression of the immunomodulatory cytokine IL10 after the liver hydrofection of the gene encoding this protein in concentrations that could have a modulatory effect. For this purpose, we employed a procedure of ‘in vivo’ liver gene injection that could be translated to clinical setting [8]. The pig model employed has a normal immune status and thus it is not possible to evaluate final immunosuppressant effect of IL10. This work represents the first confirmation that hydrofection mediates efficacious expression of an immunosuppressant protein employing a procedure that could be transferred into human clinical setting for liver transplantation.

## Methods

### Animals

The experiments were approved by the Animal Biological Research Ethics Committee of Hospital La Fe (ref. 2013/0269). All animals received humane care according to the criteria outlined in the "Guide for the Care and Use of Laboratory Animals". Pigs were individually housed in pigsties. Female pigs (18–22 kg) 3 months of age were used. Anesthesia was induced with ketamine (Imalgene^®^ 100, Merial France; 5–10 mg/kg, im), midazolam (Hospira^®^ 1 mg/ml, Madrid, Spain; 0.3 mg/kg, im) and propofol (Lipuro^®^ 2%, Braun, Melsungen, Germany; 4–6 mg/kg, iv), and was maintained with isoflurane (Isoflo^®^, Abbott laboratories, Madrid, Spain; 2.5% via the inhalation route). Muscle relaxation was induced with vecuronium bromide (Norcuron^®^ 10 mg; 0.08 mg/kg, iv). Morphine (0.4 mg/kg, iv) was administered for intraoperative analgesia, and buprenorphine (Buprex^®^, Schering-Plough, Madrid, Spain; 0.02 mg/kg, iv) was used for postoperative analgesia. Vital functions were monitored throughout the intervention to ensure the safety of the procedure, as previously reported by our group^8^. The pigs were sacrificed 1 (n = 4) or 10 (n = 5) days after the operation using potassium chloride (Braun 2 mEq, 20 mEq, iv), after sedation. Blood samples (2 ml) were collected from an ear vein at 0 h (before plasmid injection), and 1, 2, 4, 7, 10 and 14 days after injection, before sacrifice. After sacrifice, the liver was extracted, and representative tissue samples of each lobule were collected for further analysis.

### Vascular exclusion surgical procedure

The surgical procedure used three types of sutures: BiosynTM 5/0 (COVIDIEN) for vena cava, ProleneTM 6/0 (ETHICON) for portal vein, and DexonTM 2/0 (COVIDIEN) for closing the abdominal wall. Staples were used to close the skin.

To perform the transitory (7–8 min) vascular exclusion of liver by surgical procedure ‘in vivo’, a complete midline laparotomy was carried out, exposing all the abdominal organs. Liver vasculature was exposed, referenced and clamped, as previously described by our group [9]. The clamping sequence was as follows: first the hepatic artery, then the portal vein and finally the infrahepatic vena cava, to fully interrupt hepatic inflow. The suprahepatic vena cava was clamped last, to secure total hepatic vascular exclusion. A longitudinal incision was made on the anterior surface of the cava vein to insert the perfusion cannula. After gene perfusion as described in gene transfer section, the liver was kept under total vascular exclusion for no more than 5 minutes. Progressive declamping was carried out in the reverse sequence, first allowing liver outflow and finally inflow through the portal and cava veins.

### Plasmid

Plasmid p2F-hIL-10 (6.86 Kb), containing the human IL-10 protein cDNA driven by pCMV promoter, was constructed by cloning IL-10 into the HindIII site of pVITRO2 (Invitrogen, Madrid, Spain).

### ‘In vivo’ gene transfer

In this vascular exclusion surgical model, 200 ml of a solution containing the hIL10 plasmid (20 μg/ml) was injected retrogradely through suprahepatic vein at 20 ml/s whereas the other vasculature remained closed surgically.

### Quantitative PCR and RT-PCR

Tissue samples representing the whole liver were obtained 10 days after gene transfer. These samples were cut into small pieces and homogenized in buffer (Promega^®^, Barcelona, Spain) with an Ultra-Turrax homogenizer (Hielscher Ultrasonics GmbH, Teltow, Germany). Further purifications with Maxwell RNA and DNA purification from tissue kits (Promega^®^, Barcelona, Spain) were performed before spectrophotometric quantification. RNA retrotranscription to cDNA was carried out using 1 μg total RNA (DNA free), random hexamers and a High Capacity cDNA Archive Kit (Thermo Fisher, Madrid, Spain). For quantitative real-time qPCR, TaqMan PCR master mix (Thermo Fisher, Madrid, Spain) was employed according to the instructions of the manufacturer. The specific oligonucleotides for human IL-10 employed were a pre-mixed TaqMan kit from Thermo Fisher (cat no. Hs00961622_m). Quantitative data were calculated as the number of DNA and RNA copies on a regression curve which was plotted employing the same injected plasmid containing the hIL10 gene, prepared with a known concentration of hIL-10 plasmid and serial dilutions 1/10. Linearity of the standard curve included from 10^3 to 10^7 copies with a correlation coefficient > 0.95. Data plotting was performed using R (version 3.1.2) software.

### hIL-10 ELISA

The same tissue samples representing the whole liver were cut 10 days after gene transfer, and homogenized. Total protein amount was quantified using the NanoOrange protein quantitation kit (Life Technologies; CA, USA). Blood samples were collected at 0 hours, 1 hour, 1 day, 2 days, 4 days, 7 days and 10 days after gene transfer. For human IL-10 detection, BD OptEIA^®^ Human IL-10 ELISA Set (Beckton and Dickinson Biosciences, Madrid, Spain) was used following manufacturer instructions. The standard curve was prepared with hIL-10 protein. In our hands, it was a lineal standard curve from 7.8 pg/ml to 500 pg/ml with a correlation coefficient > 0.99. Results were expressed in pg/ml and were transformed to number of molecules using the protein molecular weight (20 kDa, NCBI protein database. Available: www.ncbi.nlm.nih.gov/protein/AAK38162.1). Data plotting was performed using R (version 3.1.2) software and GraphPad Prism 5 (GraphPad Software, San Diego, CA, USA).

### Molecular data expression

In order the data could be used in most of studies from different areas, the quantitative results should be referred to a common circumstance. For this reason, the data have been expressed considering a normalized cell (nc), which was classically described by Alberts et al.[10] as a typical mammalian hepatocyte with a defined content of total DNA (genome weight of a diploid cell in the specific animal, swine: 5.4 pg), RNA (20 pg) and protein (500 pg). The average volume of a cell is 2 pl. Indexes indicate the absolute number of copies of each molecular species referred to a normalized cell and they are calculated, for pig, as follows:

- Delivery index: n° transgene copies/5.4 pg of total DNA
- Transcription index: n° transgene mRNA copies/20 pg total RNA
- Translation index: n° transgene protein molecules/500 pg total protein

The efficacy of gene expression relates the protein translation index to the gene delivery index in order to define the global efficacy of the procedure. The formula referred to gene expression efficacy is defined as:

- Expression efficacy: evaluated from organ tissue or cell culture.

Expressionefficacy(proteinmoleculespergenecopy)=translationindex/deliveryindex

## Results

### Index of gene delivery

**Fig 1:**
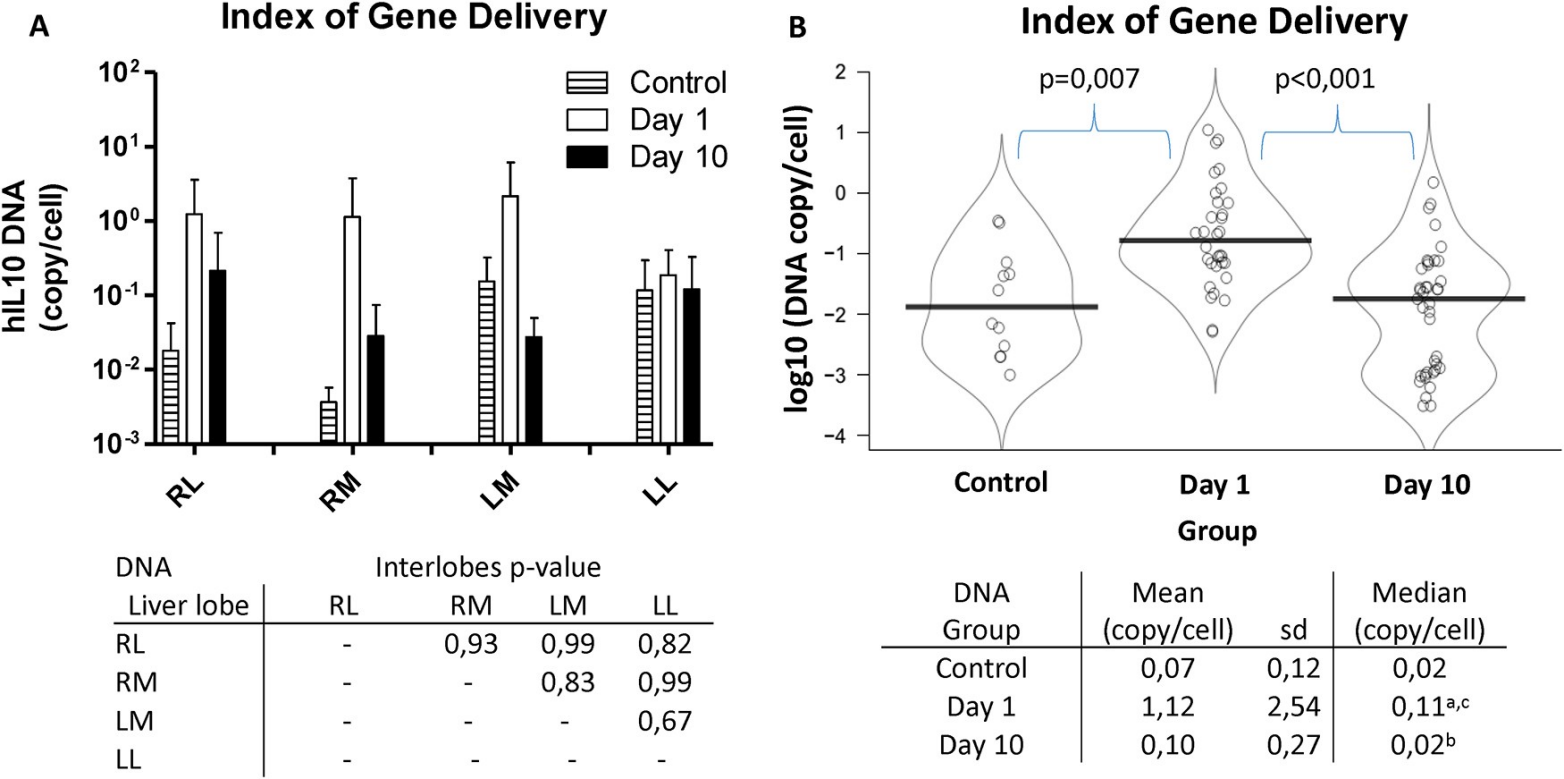
Index of gene delivery. Indexes of hIL10 DNA within liver tissue in control subjects and treated subjects at days 1 and 10 post-injection are shown, expressed as copies of hIL10 gene per cell (log10). In graph A, the average rate of DNA delivery in each liver lobe is represented. The associated table indicates the p-values obtained when comparing by pairs each liver lobe, demonstrating that no significant differences among liver lobes are observed. In graph B, the median indexes of gene delivery of control group, day 1 group and day 10 group are compared. The table associated indicates both average and median values of each group. a = group day 1 vs control, p = 0,007; b = group day 10 vs control, p = 0,679; c = group day 1 vs group day 10, p<0,001.

One and ten days after hIL10 gene transfer, pigs were sacrificed and 8 liver tissue samples (2 x 1 cm) representing the whole organ (proximal and distal vascular ileus areas of the different liver lobes: Right Lateral, RL; Right Medial, RM; Left Medial, LM; Left Lateral, LL) were collected. DNA was extracted and purified and hIL10 gene was quantified by RT-PCR. Samples from proximal and distal areas of each liver lobe were pooled and the presence of hIL10 gene was compared among liver lobes. Fig 1A shows the index of hIL10 gene delivery present in each liver lobe at days 1 and 10 after hydrofection, expressed as copy number per cell. It can be observed that index of gene delivery is similar in every liver lobe for each group, and no significant difference (p>0.6) was observed. For this reason, we pooled all the samples from each group in order to evaluate the effect of gene hydrofection at different time point with higher statistical power (Fig 1B). Control group was not treated and was employed as reference for analyze baseline. Index of gene delivery at day 1 was higher than control (p = 0.007) and day 10 group (p<0.001), achieving up to 1 copy of hIL10 gene per cell. This indicates the efficiency of gene transfection, which mediates the presence of 1 copy of gene per cell, very close to natural conditions (2 copies per diploid cell). Tissue wash out for 10 days reduced the amount of hIL10 DNA to baseline status.

### Index of gene transcription

**Fig 2:**
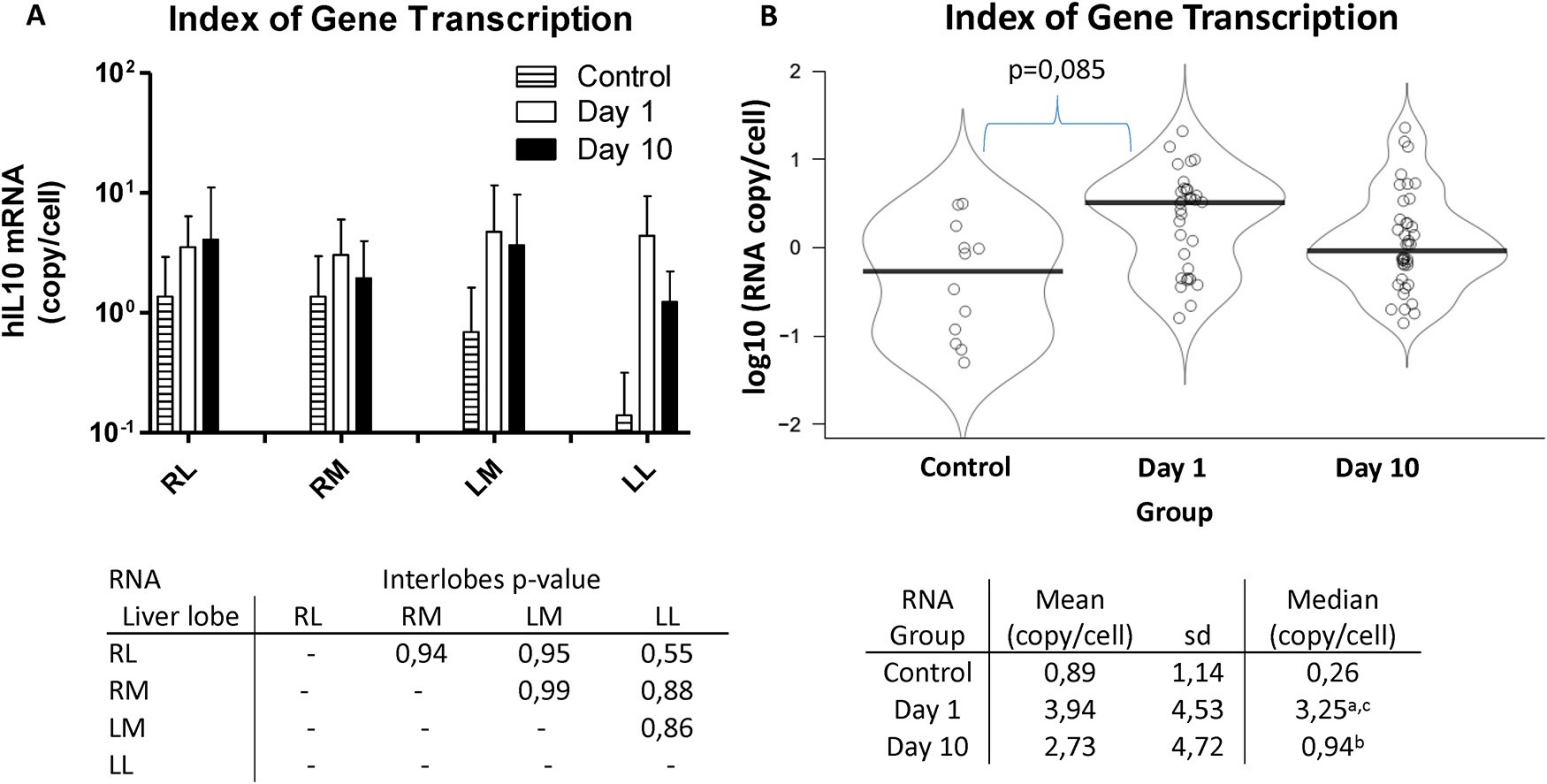
Index of gene transcription. Rates of hIL10 RNA transcription within liver tissue in control and treated groups 1 and 10 days post-injection are represented, expressed as hIL10 RNA copies per cell (log10). In graph A, the average index of RNA transcription in each liver lobe is shown. The associated table indicates the p-values obtained when comparing by pairs each liver lobe. No significant difference among liver lobes is observed. In graph B, the median indexes of gene transcription of control group, day 1 group and day 10 group are compared. The table associated reports both average and median values of each group. a = group day 1 vs control, p = 0,085; b = group day 10 vs control, p = 0,343; c = group day 1 vs group day 10, p = 0,309.

RNA samples from different liver areas were also extracted, purified and retrotranscribed to cDNA and hIL10 gene was quantified by RT-PCR. The presence of hIL10 within the same samples above indicated was compared among liver lobes. In Fig 2A, the index of hIL10 gene transcription in each liver lobe at days 1 and 10 after hydrofection is shown. Transcription index was similar in every liver lobe for each group, and no significant difference (p>0.5) was observed. For this reason, all the samples from each group were pooled in order to evaluate the gene transcription efficacy at different time points with higher statistical power. Control group pigs were not treated and were employed as reference for stablishing the expression baseline. Index of gene transcription at day 1 (3.9 copies per cell) was higher than control and diminished in day 10 (2.7 copies per cell), without reaching significant difference.

### Index of gene translation

**Fig 3:**
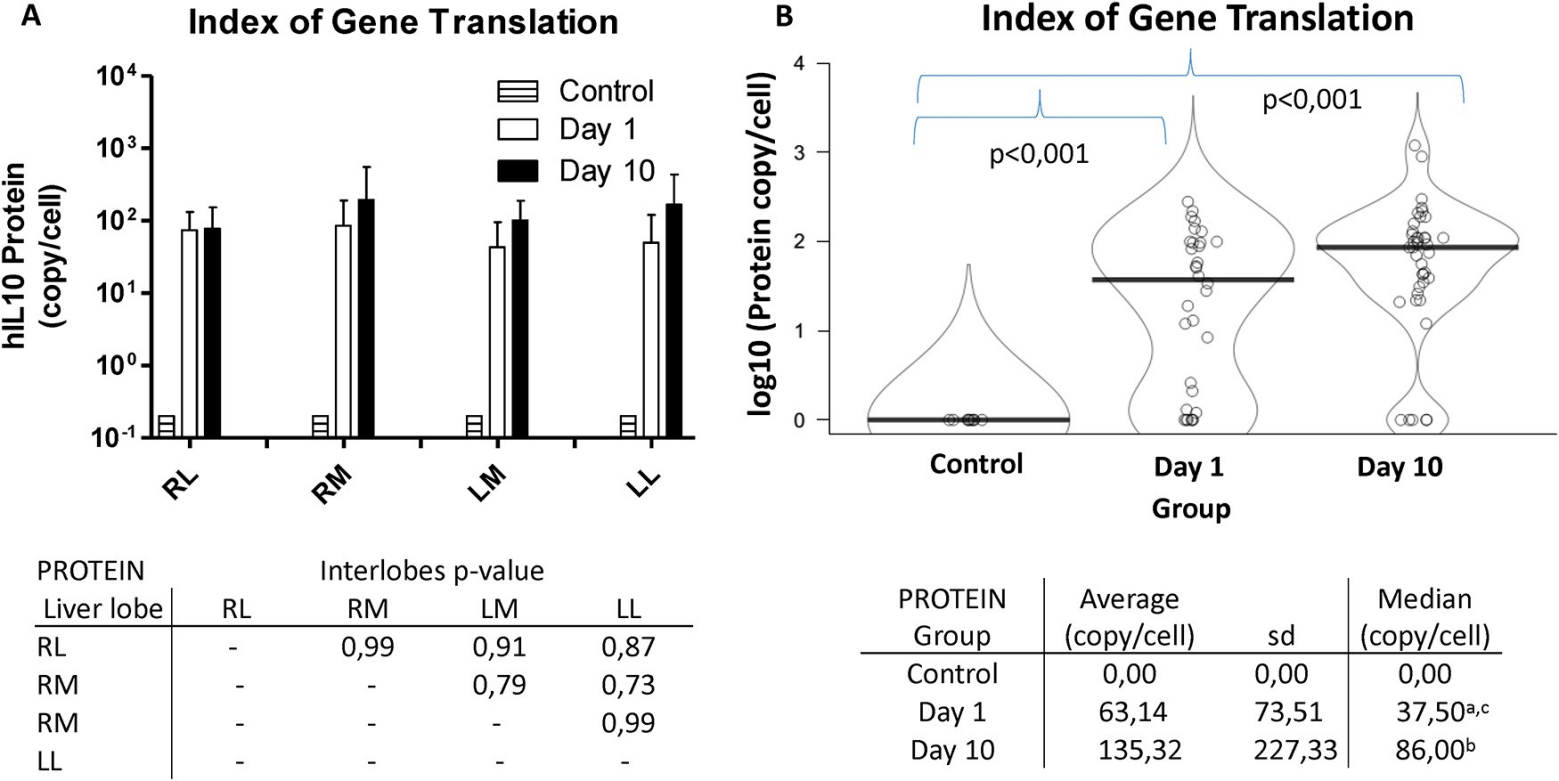
Index of gene translation. The amount of hIL10 protein within liver tissue in control and treated groups 1 and 10 days post-injection are represented. Data were expressed as hIL10 protein copies per cell (log10). In graph A, the average rate of hIL10 translation in each liver lobe is shown. The p-values obtained when comparing by pairs each liver lobe are shown in the associated table, no difference being significant. In graph B, the median indexes of gene translation of control group, day 1 group and day 10 group are compared. The table associated reports both average and median values of each group. a = group day 1 vs control, p<0,001; b = group day 10 vs control, p<0,001; c = group day 1 vs group day 10, p = 0,326.

Similar liver tissue samples representing the whole organ were collected. Total protein was extracted and purified and hIL10 protein was quantified by ELISA. Samples from proximal and distal areas of each liver lobe were pooled and the presence of hIL10 protein was compared among liver lobes. The index of hIL10 gene translation in each liver lobe at days 1 and 10 after hydrofection is shown in Fig 3A. Results were also expressed as molecules per cell. The index of gene translation was similar in every liver lobe for each group, and no significant difference was observed (p>0.7). Translation efficacy was evaluated at different time points grouping the samples from each group in order to improve statistical power. Control group pigs were not treated and were employed as reference for establishing the expression baseline. Human IL10 protein could not be detected within control pigs samples and translation index at both day 1 and 10 after transfection was significantly higher (p<0.001). Index of hIL10 gene translation increased along sampling period in treated animals and at day 10 (135.3 copies per cell) was 2-fold higher than day 1 (63 copies per cell), without reaching significant difference though.

### Gene expression efficacy

**Fig 4:**
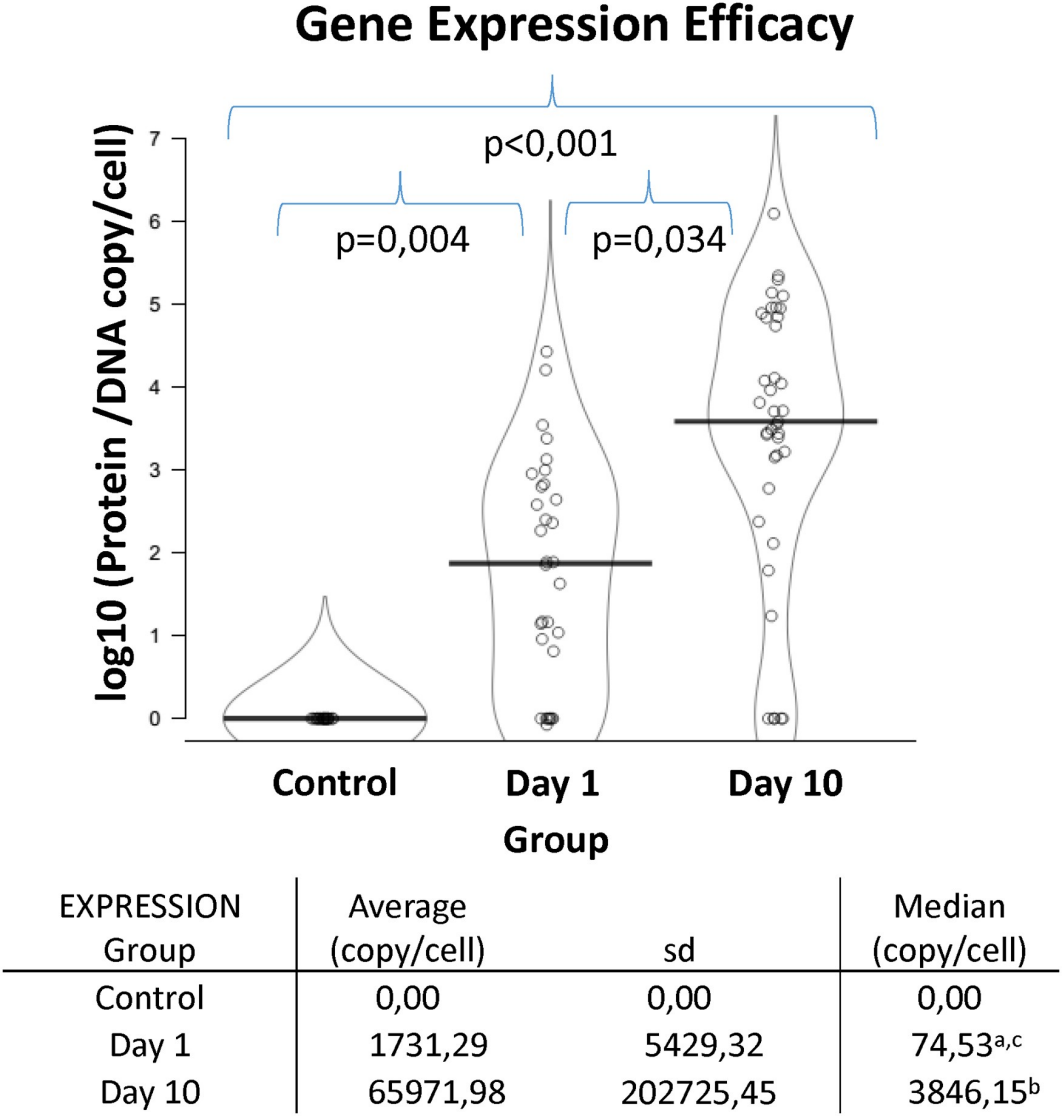
Gene expression efficacy. Indexes of gene translation and gene delivery were combined to obtain a protein/DNA ratio. The amount of hIL10 protein per copy of IL10 DNA within liver tissue in control and treated groups 1 and 10 days post-injection are represented. Data were expressed as hIL10 protein/DNA copy per cell (log10). The median values of gene expression efficacy of control group, day 1 group and day 10 group are compared. The table associated reports both average and median values of each group. a = group day 1 vs control, p = 0,004; b = group day 10 vs control, p<0,001; c = group day 1 vs group day 10, p = 0,034.

To evaluate how efficient was the protein expression of the injected gene (Fig 4), we related the indexes of gene delivery and translation (index of translation/index of delivery) of samples representing the whole organ on days 1 and 10. In samples from control non-treated pigs, the protein could not be detected and then, the expression efficacy was 0, significantly lower than the observed in treated animals both on day 1 (p = 0.004) and 10 (p<0.001). We observed that this ratio increases along time and, on day 10, the expression efficacy (>1,000 copies of protein per copy of gene) was significantly higher than on day 1 (100 copies of protein per copy of hIL10 gene; p = 0.034).

### Plasma concentration of protein

**Fig 5:**
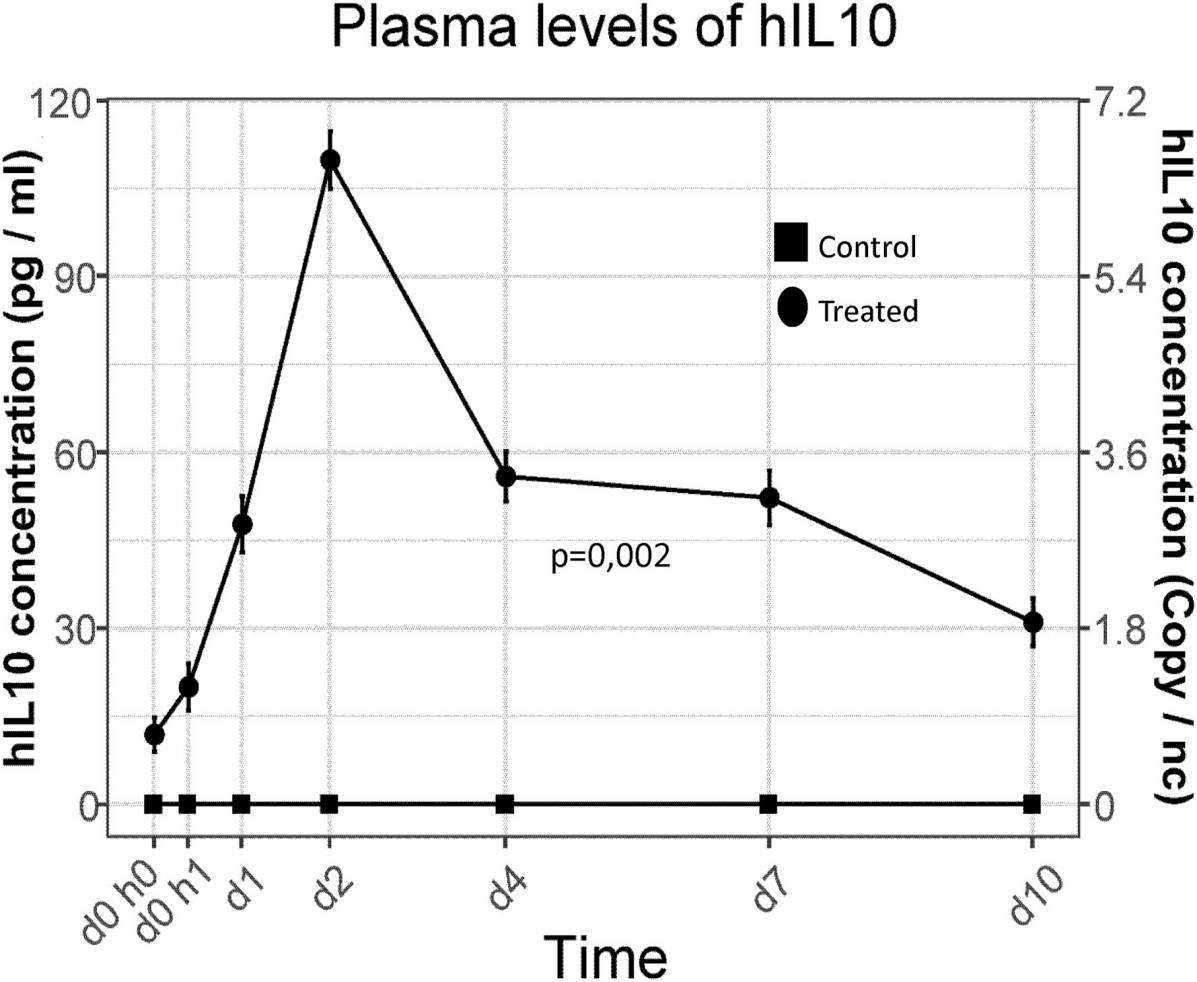
Plasma levels of hIL10 protein. The levels of hIL10 protein in peripheral blood of control and treated groups at different time points (1 hour, 1 day, 2 days, 4 days, 7 days and 10 days) after gene injection are represented. Data were expressed as hIL10 protein concentration (pg/ml, left axis) and copies per cell volume (2 pl, right axis). The average concentration of protein of control group and treated groups are compared, being significantly higher (p = 0.002) in treated groups than in control group.

Aiming to evaluate the efficacy of protein exportation, we collected peripheral blood samples from ear at different times (0h, before gene injection; 1h after injection; 1 day after injection; and 2 days, 4 days, 7 days and 10 days after injection only in those animals sacrificed 10 days after intervention). We quantified the hIL10 protein concentration in plasma (pg/ml) by ELISA. Results are shown in Fig 5. In control samples, hIL10 was undetectable. In treated animals’ samples, the protein was quantified in peripheral blood and we observed that the concentration increased very early since 1 hour after transfection, peaking on day 2 post-intervention, achieving levels of approximately 110 pg/ml of protein. From that moment, the concentration of protein decreased to approximately 50 pg/ml on days 4 and 7, and 30 pg/ml on day 10. On the right X axis, we represented the equivalence of concentration respect to the volume of a single standard cell (2 pl), expressed as protein molecules number per cell.

## Discussion

In the present study, we aimed to evaluate whether the *IL10* gene liver hydrofection could mediate amounts of cytokine in tissue and blood with potential clinical interest in liver transplantation. In absence of transplant, the “normal” immune status of the experimental pig setting made impossible to evaluate final immunosuppressant effect of IL10. Since these evaluations are of great interest, we consider performing them in future liver transplant experiments. Our results show that plasma levels of IL-10 cytokine achieved by this procedure are compatible with this subject.

Liver transplantation is the only curative procedure for those patients with end-stage liver diseases. Despite the advancements achieved in this field, there are still two important limitations that reduce the success of the strategy: the organ preservation and the host immune reaction against graft^9^.

Early mortality still occurs in 5–12% of transplanted patients, and up to 30% of whole transplanted population present acute cell rejection during the first year post-transplantation. This indicates that immune response after graft implantation is not completely controlled and should be improved. In the present study, we hypothesized that local hydrodynamic administration of a gene encoding an immunosuppressant protein could be clinically interesting since it could act as a concomitant drug and reinforce the establishment of a local immune tolerogenic status within the recipient with advantages respect to the systemic administration of recombinant proteins, which would exert pleiotropic effects. Among the different genes encoding immunosuppressant proteins, interleukin 10 was chosen due to its tolerogenic effects able to imbalance the immune equilibrium to the tolerogenic scenario at different levels that lead to Th2 immune response. Thus, a) in vitro experiments have demonstrated that exogenous IL-10 downregulates the secretion of IL-6 and tumor necrosis factor TNFα by LPS-stimulated human KCs [11], which, in turn, express more IL-10; b) IL-10 delivered by Kupffer cell decreases the expression of both MHC class II and co-stimulatory molecules expressed by LSECs [12]; c) KCs interaction with Tregs induces the secretion of IL-10 and facilitates the induction of systemic tolerance to hepatocyte-derived antigens [13]; d) IL-10 produced by KCs and Treg protects the liver from injury induced by concavalin A [14]; e) The liver cytokine milieu (including IL-10 and TGF-β), induced by the complex interplay of KCs, LSECs, HSCs and other cell composites, can cause hepatic DCs to become tolerogenic [15–21].

In order to optimize the efficiency of *IL10* gene liver hydrofection, we employed a vascular exclusion surgical procedure that permitted pressurizing the organ in a porcine in vivo model since it has been previously described that vascular exclusion increases the efficacy of liver gene delivery [22,23]. This procedure has proved to be safe in large animals as dogs [24] and pigs by both histological analysis [8] (including TEM tissue preparations [9]), that do not show structural damage, and biochemical determination of liver enzymes [9,24], which rapidly normalize to basal levels. Another study [24] evaluated the effect of liver hydrofection on cytokines expression in dogs. It was reported the increase of TNF-α and IL-10 2 hours after hydrofection but these normalized to basal levels 24 hours after the intervention.

In this study, we evaluate the efficacy of hydrodynamic gene transfer by quantifying the indexes of gene delivery, transcription and translation both in tissue and peripheral blood, 1 and 10 days after the intervention, which were expressed as copy number of each molecular specie per normalized cell. The gene delivery index 1 day after hydrofection reached 1 copy/10 nc of hIL10 gene, this rate decreasing on day 10 to levels close to those considered technique detection zero. This DNA was efficiently transcribed and each copy of gene lead to 10 copies of RNA. Regarding the protein production, we observed translation rates of up to 100 copies/nc in liver tissue that increased from day 1 to day 10. This protein produced was efficiently released to bloodstream, where it can exert its multiple functions mediating protein levels in peripheral blood of more than 100 pg/ml, being 124 pg/ml the IC50 of interleukin 10 for TNFα inhibitory effect [25]. Considering that samples were collected from ear vein, we could expect that blood concentrations in liver area should be higher, permitting a local higher effect. This procedure proved to be safe, since none of the animals suffered from adverse reactions derived from neither the surgical intervention nor the drug administered.

The results observed in this work are in agreement with those reported in a previous article of our group [9] with IL10 gene transfer in human liver segments, supporting the efficiency of hydrofection for gene transfer and decoding and its potential to be translated into clinical setting, mainly because this procedure of liver hydrofection by surgical vascular exclusion could be performed by minimally invasive strategies of interventionist catheterization [8, 26].

Further studies employing entire human livers, preserved in a normothermic perfusion system, could help to determine the efficacy of gene translation and protein release in human tissue maintained under physiological conditions. However, the implementation (and efficiency) of the procedure in an experimental model of liver transplantation in swine, transfecting the IL10 gene in the transplanted organ prior to its implantation in the recipient remain to be unknown and could be the final translational step prior to its evaluation in a clinical trial. This model would also permit evaluating the final functional effects of IL-10 on the expression on other immune-related genes and molecules. The procedure is totally transferrable to clinical setting. Previous work by Tsoulfas et al. [27] performed hydrodynamic liver gene transfer in a liver transplant setting employing a rat model and efficient liver function and gene expression was reported. Gene was transferred to the liver within the cold ischemia period between organ extraction from donor and organ implantation. We agree that the optimal moment for gene transfer, regarding surgical technic, time and transfection process, would be after donor liver bank surgery prior to its implantation in recipient. This would affect minimally the liver transplantation procedure and gene transfection could be performed controlling all the parameters (complete vascular exclusion, flow rate, pressure). Furthermore, very often the graft is obtained in hospitals different to where the recipient is transplanted and this protocol would permit the procedure reproducibility. Also, in these pre-clinical models, safer minicircular gene constructions could be employed since they offer potential translational advantages, such as elimination of bacterial genetic sequences, smaller size to facilitate and improve its delivery and higher final expression of the protein.

## Supporting information

S1 Table Raw data of *hIL10* gene delivery from each sample and group (control non-treated, day 1 and day 10) are presented. Tissue levels of DNA are expressed as copy number per cell according to normalized values of an average mammalian cell (5.4 pg of DNA). Data from pig samples shown correspond to different liver areas: RL (right lateral), RM (right medial), LM (left medial), LL (left lateral). 1 means proximal and 2 means distal. NA: non detected.
(XLSX)

S2 Table Raw data of *hIL10* gene transcription from each sample and group (control non-treated, day 1 and day 10) are presented. Tissue levels of RNA are expressed as copy number per cell according to normalized values of an average mammalian cell (20 pg of RNA). Data from pig samples shown correspond to different liver areas: RL (right lateral), RM (right medial), LM (left medial), LL (left lateral). 1 means proximal and 2 means distal. NA: non detected.
(XLSX)

S3 Table Raw data of hIL10 protein translation from each sample and group (control non-treated, day 1 and day 10) are presented. Tissue levels of protein are expressed as copy number per cell according to normalized values of an average mammalian cell (500 pg of protein). Data from pig samples shown correspond to different liver areas: RL (right lateral), RM (right medial), LM (left medial), LL (left lateral). 1 means proximal and 2 means distal. NA: non detected.
(XLSX)

S4 Table Human IL10 protein concentration in plasma. Plasma levels of hIL10 protein are expressed as concentration units (pg/ml). NA: non detected.
(XLSX)

S5 Table Raw data values of gene expression efficacy. This corresponds to the interleukin 10 protein/DNA copies ratio per cell. Data from pig samples shown correspond to different liver areas: RL (right lateral), RM (right medial), LM (left medial), LL (left lateral). 1 means proximal and 2 means distal. NA: non detected.
(XLSX)

## Abbreviations

CD4: cluster of differentiation 4
CD8: cluster of differentiation 8
cDNA: complementary deoxyribonucleic acid
DCs: dendritic cells
DNA: deoxyribonucleic acid
ELISA: enzyme linked immunosorbent assay
GM-CSF: granulocyte/monocyte colony stimulating factor
HSCs: Hematopoietic stem cells
IC_50_: concentration for 50% inhibition
IL: Interleukin
KCs: Kupffer cells
LPS: Lipopolysaccharides
LSECs: Liver sinusoidal endothelial cells
NK: natural killer
NKT: natural killer T cells
pCMV: Cytomegalovirus promoter
RNA: ribonucleic acid
RT-PCR: real time polymerase chain reaction
TGFβ: transforming growth factor beta
TNFα: Tumor necrosis factor alpha
Treg: regulatory T cells
